# Correction: Injectable hydrogel-based localized delivery of IDO-galectin-3 mitigates neuroinflammation and promotes neuronal sparing after spinal cord contusion in rats

**DOI:** 10.1039/d6tb90064j

**Published:** 2026-05-20

**Authors:** Gopal Agarwal, Allison Campbell, Arun Wanchoo, Daniel Plant, Izabela Zmirska, Jacob Fuhr, Prodip Bose, Gregory A. Hudalla, Benjamin G. Keselowsky, Christine E. Schmidt

**Affiliations:** a J. Crayton Pruitt Family Department of Biomedical Engineering, Herbert Wertheim College of Engineering, University of Florida Gainesville FL 32610 USA bkeselowsky@bme.ufl.edu schmidt@bme.ufl.edu; b Department of Anesthesiology and Department of Neurology, College of Medicine, University of Florida Gainesville Florida 32610 USA; c Brain Rehabilitation Research Center, Malcom Randall Veterans Administration Medical Center Gainesville Florida 32610 USA

## Abstract

Correction for ‘Injectable hydrogel-based localized delivery of IDO-galectin-3 mitigates neuroinflammation and promotes neuronal sparing after spinal cord contusion in rats’ by Gopal Agarwal *et al.*, *J. Mater. Chem. B*, 2026, **14**, 3464–3479, https://doi.org/10.1039/D5TB02228B.

The authors regret that a project funder was omitted from the Acknowledgements section in the published article. The corrected Acknowledgements section should read as follows.

The authors would like to acknowledge the funding support received from NIH National Institute of Neurological Disorders and Stroke (grant number 1R21NS123596-01) and NIH National Center for Medical Rehabilitation Research T32 Neuromuscular Plasticity Training Program (grant number T32HD043730).

The Royal Society of Chemistry apologises for these errors and any consequent inconvenience to authors and readers.

